# Allergic Rhinitis and Allergic Sensitization in Pediatric Otitis Media with Effusion: A Systematic Review and Meta-Analysis with Narrative Synthesis of Eustachian Tube Dysfunction

**DOI:** 10.3390/children13070892

**Published:** 2026-07-03

**Authors:** Alina-Mihaela Petre, Romeo Costin, Ion Anghel

**Affiliations:** 1 Department of Anatomy, Carol Davila University of Medicine and Pharmacy, 050474 Bucharest, Romania; 2 “Dr. Nicolae Kretzulescu” Outpatient Diagnostic and Treatment Medical Center, 050042 Bucharest, Romania; 3 Department of Otorhinolaryngology, Carol Davila University of Medicine and Pharmacy, 050474 Bucharest, Romania; romeo.costin@umfcd.ro (R.C.); ion.anghel@umfcd.ro (I.A.); 4 ENT Clinical Department, “Dr. Carol Davila” Central Military Emergency University Hospital, 010825 Bucharest, Romania

**Keywords:** allergic rhinitis, atopy, IgE sensitization, otitis media with effusion, Eustachian tube dysfunction, tympanometry, middle-ear dysfunction, children, meta-analysis, systematic review

## Abstract

**Highlights:**

**What are the main findings?**

- Allergic rhinitis/rhinitis was significantly associated with pediatric otitis media with effusion, with a pooled odds ratio of 3.73 in the primary random-effects meta-analysis.
- Atopy or IgE sensitization was also associated with OME, but the evidence was based on fewer studies and showed high heterogeneity.

**What are the implications of the main findings?**

- Allergic rhinitis and allergic sensitization should be considered potential contributing factors in selected pediatric OME phenotypes, especially when persistent nasal symptoms coexist with ear complaints, hearing concerns, or abnormal tympanometry.
- The findings support phenotype-based ENT and allergy/immunology assessment, but do not justify treating all pediatric OME as allergy-driven disease.

**Abstract:**

Background: Otitis media with effusion (OME) is a common pediatric middle-ear disorder with multifactorial pathogenesis. Allergic rhinitis and allergic sensitization have been proposed as contributing factors, but previous evidence remains heterogeneous. This systematic review and meta-analysis aimed to clarify the association between allergic rhinitis, allergic sensitization, and pediatric OME, while separately considering Eustachian tube dysfunction (ETD) and middle-ear dysfunction. Methods: A systematic search was conducted in PubMed/MEDLINE, Scopus, and the Cochrane Library. Observational studies evaluating allergic rhinitis, rhinitis, atopy, IgE sensitization, or allergy-related symptoms in relation to OME, ETD, abnormal tympanometry, or middle-ear dysfunction were considered eligible. Random-effects meta-analyses were performed separately for allergic rhinitis/rhinitis–OME and atopy/IgE sensitization–OME. ETD and middle-ear dysfunction outcomes were synthesized narratively because of methodological heterogeneity. Results: After removal of duplicates, 956 records were screened, 46 reports were assessed for eligibility, and 21 studies were included in the qualitative or narrative synthesis. Nine studies contributed to at least one pooled quantitative synthesis, while one additional birth-cohort study contributed adjusted estimates to the narrative sensitivity interpretation. Allergic rhinitis/rhinitis was significantly associated with OME in the primary meta-analysis (OR = 3.73, 95% CI 1.79–7.74, *p* < 0.001; I^2^ = 69.7%). The association remained significant in an expanded sensitivity analysis including one additional community-based study (OR = 3.46, 95% CI 1.92–6.23, *p* < 0.001; I^2^ = 63.7%). Atopy/IgE sensitization was also associated with OME (OR = 5.45, 95% CI 1.33–22.35, *p* = 0.018; I^2^ = 86.0%), although this analysis included only two studies. Most pooled estimates were derived from crude data and should therefore be interpreted as associations rather than causal effects. Conclusions: Allergic rhinitis/rhinitis and allergic sensitization are associated with increased odds of pediatric OME. However, the available evidence is mainly observational and heterogeneous. Allergic disease should be considered a potential contributing factor in selected pediatric OME phenotypes, rather than a universal or isolated cause. A phenotype-based approach involving both otolaryngology and allergy/immunology may be clinically useful.

## 1. Introduction

Otitis media with effusion (OME) is a common pediatric middle-ear disorder defined by the presence of non-purulent fluid behind an intact tympanic membrane, without signs or symptoms of acute infection [1,2]. It represents one of the most frequent causes of acquired conductive hearing loss in childhood and may affect speech development, school performance, and quality of life when persistent or recurrent [1]. Although many episodes resolve spontaneously, persistent OME remains clinically important because it may require audiological monitoring, medical follow-up, or surgical intervention in selected cases [1,2].

The pathogenesis of OME is multifactorial. Eustachian tube dysfunction (ETD), recurrent upper respiratory tract infections, adenoid hypertrophy, immature immune responses, environmental exposures, and host susceptibility have all been implicated [2,3]. Among these potential contributors, allergic rhinitis and allergic sensitization have attracted long-standing interest because of the anatomical and immunological continuity between the nose, nasopharynx, Eustachian tube, and middle-ear cavity [3,4,5]. Allergic inflammation may promote nasopharyngeal mucosal edema, impaired mucociliary clearance, altered mucus properties, and functional obstruction of the Eustachian tube, thereby contributing to negative middle-ear pressure and effusion formation [4,5].

However, the relationship between allergic disease and OME remains controversial. Previous studies have used heterogeneous definitions of allergic rhinitis, nasal allergy, atopy, IgE sensitization, and middle-ear outcomes, which complicates interpretation and quantitative synthesis [1,2,3]. Some studies have reported a high prevalence of allergic rhinitis or atopy among children with chronic or recurrent OME, whereas others have found weak or non-significant associations [1,2]. Moreover, allergic rhinitis and allergic sensitization are often treated as interchangeable exposures, despite being clinically distinct constructs. Allergic rhinitis implies symptomatic nasal disease related to allergen exposure, whereas atopy or IgE sensitization may exist without clinically relevant rhinitis.

Previous meta-analytic evidence suggested that allergic rhinitis and allergy are risk factors for OME [6]. Nevertheless, several limitations remain relevant for current clinical interpretation. First, allergic rhinitis, nonspecific rhinitis, atopy, and IgE sensitization are frequently pooled together despite differences in diagnostic meaning. Second, OME, ETD, abnormal tympanometry, and middle-ear pressure abnormalities are sometimes discussed together, although they are not equivalent outcomes. Third, newer studies have become available, including community-based, birth-cohort, and ETD-focused studies, which justify an updated synthesis.

The ETD literature is particularly relevant from an otolaryngological perspective. Recent reviews suggest that allergic mechanisms may contribute to ETD through Th2-mediated inflammation, mucosal swelling, impaired clearance, surfactant alterations, and extension of nasopharyngeal inflammation toward the tubal mucosa [4]. At the same time, experimental evidence indicates that allergen exposure alone may be insufficient to induce OME, supporting a more nuanced model in which allergy acts as one contributor among several interacting mechanisms rather than as a universal isolated cause [5,7].

Therefore, this systematic review and meta-analysis aimed to update and refine the evidence regarding the association between allergic rhinitis, allergic sensitization, and pediatric OME. We specifically separated clinically defined allergic rhinitis/rhinitis from atopy or IgE sensitization and treated ETD and middle-ear dysfunction as secondary narrative outcomes because of methodological heterogeneity. By doing so, we aimed to provide a synthesis that is more directly useful for pediatric ENT and allergy practice.

## 2. Materials and Methods

### 2.1. Study Design and Reporting

This systematic review and meta-analysis was conducted in accordance with the Preferred Reporting Items for Systematic Reviews and Meta-Analyses 2020 statement [8]. The review was designed to evaluate the association between allergic rhinitis, allergic sensitization, and otitis media with effusion (OME), with a secondary narrative synthesis of Eustachian tube dysfunction (ETD) and broader middle-ear dysfunction outcomes (Supplementary File S1).

The primary quantitative analysis focused on the association between allergic rhinitis/rhinitis and OME. An exploratory quantitative analysis evaluated the association between atopy or IgE sensitization and OME. ETD and middle-ear dysfunction outcomes were analyzed narratively because the available studies used heterogeneous definitions, populations, and diagnostic methods.

### 2.2. Eligibility Criteria

For the primary OME analyses, eligible populations were children and adolescents. Adult or mixed-age studies were eligible only for the predefined secondary narrative synthesis of Eustachian tube dysfunction or broader middle-ear dysfunction and were not pooled with pediatric OME studies. Eligible studies reported data on allergic rhinitis, rhinitis, nasal allergy, atopy, IgE sensitization, or allergy-related symptoms in relation to OME, ETD, abnormal tympanometry, altered middle-ear pressure, or related middle-ear dysfunction outcomes.

Eligible designs included observational cohort, case–control, cross-sectional, school-screening, community-based, and population-based studies. Studies were considered for quantitative synthesis when they provided extractable 2 × 2 data or effect estimates allowing calculation or interpretation of odds ratios.

Studies that were clinically relevant but unsuitable for pooling were retained for qualitative or narrative synthesis only when they provided a distinct, sufficiently detailed, and methodologically interpretable contribution to the predefined narrative outcomes. Non-comparative, treatment-response, or mechanistic reports lacking sufficiently verifiable methods, outcomes, or a unique contribution were excluded.

Studies were excluded if they were reviews, editorials, case reports, animal or in vitro studies, studies focused exclusively on acute otitis media, studies without an allergy-related exposure, studies without an OME/ETD-related outcome, duplicate or overlapping cohorts, reports without verifiable bibliographic details, or studies lacking sufficient data for either qualitative or quantitative synthesis. Abstract-only records for which a sufficiently detailed full report could not be obtained were excluded because eligibility, methodological quality, and outcome reporting could not be adequately assessed.

### 2.3. Information Sources and Search Strategy

A systematic literature search was performed in PubMed/MEDLINE, Scopus, and the Cochrane Library. Manual screening of relevant references and eligible articles was also performed.

The search strategy combined terms related to allergic disease and sensitization with terms related to OME and ETD. Search concepts included allergic rhinitis, rhinitis, allergy, atopy, IgE sensitization, otitis media with effusion, middle-ear effusion, Eustachian tube dysfunction, tympanometry, and middle-ear dysfunction.

No date restriction was applied during the initial search. Records were imported into a screening workbook, and duplicate records were removed before title and abstract screening. The final search was performed on 8 June 2026. The complete search strategy is provided in Supplementary Table S1. A formal review protocol was not prospectively registered or made publicly available; eligibility, extraction, and synthesis procedures were defined in the working review plan before the final analyses.

### 2.4. Study Selection

After deduplication, records were screened by title and abstract. Reports considered potentially relevant were sought for retrieval and assessed for eligibility. Studies were categorized as eligible for quantitative synthesis, eligible for qualitative or narrative synthesis only, excluded after eligibility assessment, duplicate or overlapping cohort, or not retrievable. Screening and eligibility assessment were performed by the first author, with uncertain records discussed with the senior authors.

When multiple reports appeared to describe overlapping populations or closely related datasets, only the most complete or methodologically suitable report was retained for synthesis. Reports with insufficient bibliographic information or unverifiable source details were excluded.

### 2.5. Data Extraction

Data were extracted by the first author (A.M.P.) using a predefined extraction form. Unclear or ambiguous information was discussed with the senior authors before final categorization. For each eligible study, the following data were extracted: first author, year of publication, country, study design, population characteristics, age group, sample size, exposure definition, outcome definition, diagnostic methods, comparator group, 2 × 2 data where available, reported odds ratios, adjusted odds ratios where available, and relevant methodological notes.

For quantitative synthesis, data were extracted using the following convention:

- a = exposed participants with outcome;
- b = exposed participants without outcome;
- c = unexposed participants with outcome;
- d = unexposed participants without outcome.

Individual odds ratios were calculated as:

OR = (a × d)/(b × c).

Allergic rhinitis/rhinitis studies were extracted separately from atopy or IgE sensitization studies. This separation was prespecified to avoid pooling clinically expressed allergic rhinitis with immunological sensitization alone.

### 2.6. Outcomes

The primary outcome was the association between allergic rhinitis/rhinitis and OME.

The exploratory quantitative outcome was the association between atopy or IgE sensitization and OME.

Additional secondary outcomes included ETD and middle-ear dysfunction, including abnormal tympanometry, altered middle-ear pressure, ETDQ-7 abnormalities, tubomanometry findings, mastoid pneumatization abnormalities, or otologic history. These outcomes were synthesized narratively.

### 2.7. Risk of Bias Assessment

Risk of bias was assessed independently by two reviewers (A.M.P. and R.C.) for the 10 comparative studies contributing to the quantitative syntheses or adjusted sensitivity interpretation, using design-specific Joanna Briggs Institute critical appraisal tools. Disagreements were resolved through discussion. Narrative-only mechanistic, descriptive, non-comparative, and treatment-response studies were used for contextual interpretation and were not included in this formal JBI appraisal because they did not contribute comparative effect estimates to the quantitative evidence base. The JBI checklist for analytical cross-sectional studies was applied to cross-sectional and school-screening studies, including the cross-sectional analysis conducted within the prospective birth cohort, whereas the JBI checklist for case–control studies was applied to case–control studies. The appraisal considered participant selection, comparability of groups, validity and reliability of exposure and outcome measurement, identification and management of confounding, and appropriateness of statistical analysis. Each item was rated as “Yes”, “No”, “Unclear”, or “Not applicable”. Overall judgments of low, moderate, moderate-to-high, or high risk of bias were based on the methodological importance of the identified limitations rather than on an unweighted numerical score. The detailed item-level assessments are provided in Supplementary Table S3.

### 2.8. Statistical Analysis

For studies providing extractable 2 × 2 data, crude odds ratios and 95% confidence intervals were calculated. Data were extracted using a consistent convention: a = exposed participants with outcome, b = exposed participants without outcome, c = unexposed participants with outcome, and d = unexposed participants without outcome. Individual odds ratios were calculated as:

OR = (a × d)/(b × c).

Meta-analyses were performed on log odds ratios using inverse-variance weighting under a random-effects model, because clinical and methodological heterogeneity was expected across studies. Heterogeneity was quantified using the I^2^ statistic. No continuity correction was required because none of the included 2 × 2 tables contained zero cells.

The primary meta-analysis pooled studies evaluating allergic rhinitis/rhinitis and OME. An expanded sensitivity analysis additionally included one recent community-based study using a broader clinical symptom-based definition of allergic rhinitis. This study was considered separately because allergic rhinitis did not remain an independent predictor of OME in multivariable analysis.

An exploratory random-effects meta-analysis pooled studies evaluating atopy or IgE sensitization and OME. Studies reporting adjusted odds ratios without extractable 2 × 2 data were not pooled directly with crude odds ratios in the main analysis but were considered in narrative or sensitivity interpretation. A separate generic inverse-variance synthesis of adjusted estimates was not performed because only a small number of studies reported adjusted associations, and these studies differed substantially in exposure definition, study design, population, OME ascertainment, and covariate adjustment, precluding a clinically meaningful pooled estimate.

ETD and broader middle-ear dysfunction outcomes were not pooled quantitatively because of heterogeneity in definitions, diagnostic methods, and outcome measures. These outcomes were synthesized narratively. Formal small-study-effect or publication-bias testing was not performed because the number of studies in each quantitative synthesis was small. Calculations were performed using a predefined extraction workbook based on the extracted 2 × 2 data, and forest plots were generated from the study-level effect estimates.

## 3. Results

### 3.1. Search Results and Study Selection

**Figure 1:**
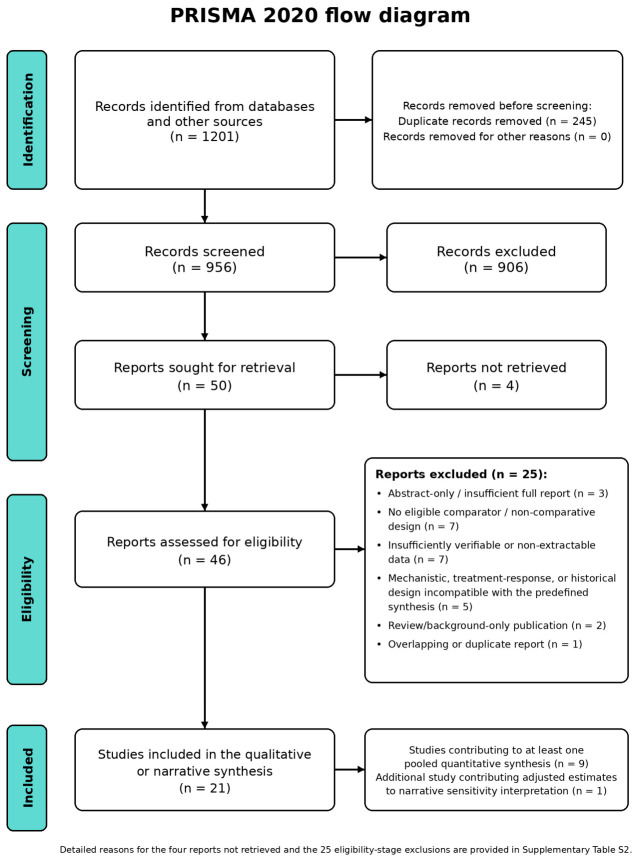
PRISMA 2020 flow diagram of study selection [8].

The database and manual searches identified 1201 records. After removal of 245 duplicates, 956 records were screened by title and abstract. Of these, 906 records were excluded as clearly irrelevant, leaving 50 reports sought for retrieval. Four reports could not be retrieved. Therefore, 46 reports were assessed for eligibility. After eligibility assessment, 25 reports were excluded for reasons including absence of an eligible comparator, lack of extractable data, duplicate or overlapping reports, non-eligible outcomes, review, discussion, or background-only publication, abstract-only reporting with insufficient methodological information, or insufficiently verifiable bibliographic information. Overall, 21 studies were included in the qualitative or narrative synthesis. Nine studies contributed to at least one pooled quantitative synthesis, while one additional birth-cohort study contributed adjusted estimates to the narrative sensitivity interpretation. The study-selection process is presented in Figure 1.

The diagram summarizes the identification, screening, eligibility assessment, and inclusion of studies in the systematic review and meta-analysis. Background-only references used solely for contextual discussion were not counted as included studies.

### 3.2. Characteristics of Included Studies

The studies included in the quantitative synthesis were published between 1998 and 2024. Additional qualitative and mechanistic studies used for narrative interpretation covered a broader period, from 1984 to 2024. The included studies were predominantly observational and included cross-sectional, case–control, prospective case–control, birth cohort, school-screening, community-based, and nationally representative cross-sectional designs.

Most studies focused on pediatric populations. Adult or mixed-age studies were retained only when they were relevant to Eustachian tube dysfunction or broader middle-ear dysfunction. The main quantitative synthesis evaluated the association between allergic rhinitis/rhinitis and otitis media with effusion. Six studies contributed to the primary allergic rhinitis/rhinitis–OME meta-analysis [9,10,11,12,13,14]. One additional community-based study was included in an expanded sensitivity analysis because allergic rhinitis was clinically defined and the adjusted association was not statistically significant [15]. Two studies contributed to the exploratory atopy/IgE sensitization–OME meta-analysis [16,17].

OME was most commonly assessed using otoscopy and tympanometry. Some studies used type B tympanograms as the main objective marker, whereas others also considered type C tympanograms, negative middle-ear pressure, absent acoustic reflexes, or persistent effusion criteria. Allergic rhinitis was variably defined using clinical symptoms, physician assessment, nasal findings, questionnaires, or combinations of symptoms and allergy-related history. Atopy and IgE sensitization were assessed more objectively, typically through skin-prick testing, CAP-FEIA, or specific IgE testing.

Studies addressing Eustachian tube dysfunction or middle-ear dysfunction used heterogeneous outcomes, including ETDQ-7 scores, tympanometry, tubomanometry, middle-ear pressure, abnormal tympanogram patterns, mastoid pneumatization, and otologic history. Because of this heterogeneity, these studies were synthesized narratively rather than pooled quantitatively.

The characteristics of included studies are summarized in Table 1.

### 3.3. Primary Meta-Analysis: Allergic Rhinitis/Rhinitis and OME

Six studies were included in the primary random-effects meta-analysis evaluating allergic rhinitis/rhinitis and OME [9,10,11,12,13,14]. The pooled analysis showed that allergic rhinitis/rhinitis was significantly associated with increased odds of OME:

OR = 3.73, 95% CI 1.79–7.74, *p* < 0.001; I^2^ = 69.7%.

The direction of effect generally favored a positive association between allergic rhinitis/rhinitis and OME. Individual study effects varied, and the observed heterogeneity likely reflected differences in exposure definitions, OME diagnostic criteria, study populations, recruitment settings, and control of confounding rather than the influence of a single study.

**Figure 2:**
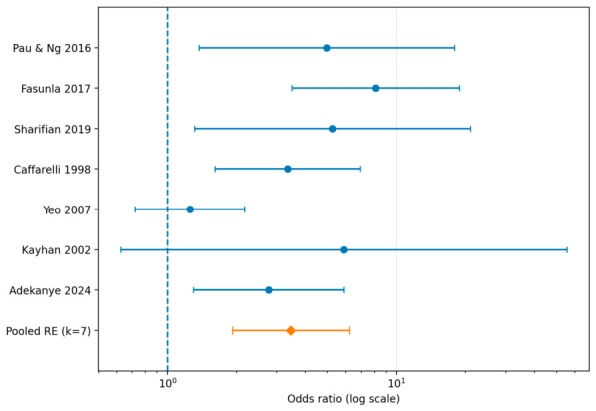
Forest plot of the association between allergic rhinitis/rhinitis and pediatric otitis media with effusion, including the expanded sensitivity analysis [9,10,11,12,13,14,15]. Blue points and horizontal lines represent study-specific odds ratios and 95% confidence intervals. The orange diamond and horizontal line represent the pooled random-effects estimate and its 95% confidence interval. The dashed vertical line indicates the null effect (OR = 1). Odds ratios greater than 1 indicate higher odds of OME among children with allergic rhinitis/rhinitis. CI, confidence interval; OME, otitis media with effusion; OR, odds ratio.

Individual study-level effect estimates and 2 × 2 data are shown in Table 2. Study-level estimates are displayed in Figure 2, together with the expanded sensitivity model; the primary pooled estimate is reported in Table 2.

### 3.4. Expanded Sensitivity Analysis

An expanded sensitivity analysis was performed by adding one recent community-based study that used a clinical symptom-based definition of allergic rhinitis [15]. In this study, children with allergic rhinitis had a higher prevalence of OME than children without allergic rhinitis; however, allergic rhinitis did not remain an independent predictor of OME in multivariable analysis [15]. Therefore, this study was not used as a primary driver of the main pooled estimate but was included in an expanded sensitivity model.

After inclusion of this additional study, the association remained statistically significant:

OR = 3.46, 95% CI 1.92–6.23, *p* < 0.001; I^2^ = 63.7%.

Compared with the primary analysis, the pooled effect changed only modestly, and heterogeneity decreased from 69.7% to 63.7%. The limited change in the crude pooled estimate suggests numerical stability of the unadjusted association; however, the absence of an independent adjusted association in Adekanye et al. indicates that confounding may partly account for the observed relationship and prevents this sensitivity analysis from being interpreted as confirmatory evidence.

### 3.5. Exploratory Quantitative Synthesis of Atopy/IgE Sensitization and OME

Two studies were included in the exploratory random-effects meta-analysis evaluating atopy or IgE sensitization and OME [16,17]. This analysis was kept separate from the allergic rhinitis/rhinitis analysis because allergic sensitization and clinically expressed allergic rhinitis are related but distinct constructs.

The pooled analysis showed a significant association between atopy/IgE sensitization and OME:

OR = 5.45, 95% CI 1.33–22.35, *p* = 0.018; I^2^ = 86.0%.

This result suggests that allergic sensitization may be associated with increased odds of OME. However, the finding should be interpreted cautiously because the analysis included only two studies and heterogeneity was high.

**Figure 3:**
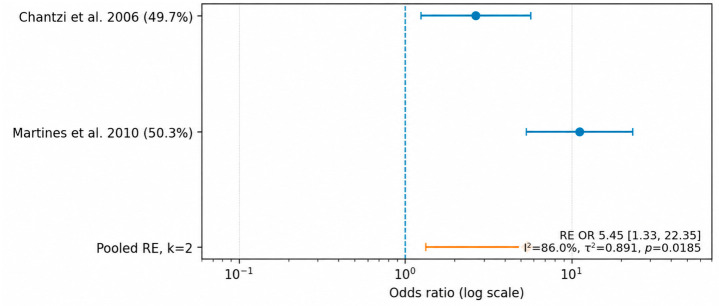
Forest plot of the association between atopy/IgE sensitization and pediatric otitis media with effusion [16,17]. Blue points and horizontal lines represent study-specific odds ratios and 95% confidence intervals. The orange marker and horizontal line represent the pooled random-effects estimate and its 95% confidence interval. The dashed vertical line indicates the null effect (OR = 1). CI, confidence interval; IgE, immunoglobulin E; OME, otitis media with effusion; OR, odds ratio; RE, random effects.

The study-level data and pooled estimate are shown in Table 2, and the forest plot is presented in Figure 3.

### 3.6. Narrative Synthesis of Eustachian Tube Dysfunction and Middle-Ear Dysfunction

The evidence regarding allergic rhinitis, ETD, and middle-ear dysfunction was synthesized narratively because the included studies used heterogeneous populations, diagnostic tools, and outcomes. Some studies assessed Eustachian tube function using ETDQ-7, tympanometry, tubomanometry, or middle-ear pressure measurements, while others evaluated abnormal tympanograms, otologic history, tympanostomy tube placement, or mastoid pneumatization.

Overall, the ETD and middle-ear dysfunction literature supported biological plausibility for a relationship between allergic rhinitis or atopy and altered middle-ear physiology. Controlled and observational studies suggested that allergic rhinitis may be associated with abnormal Eustachian tube function, negative middle-ear pressure, abnormal tympanometric patterns, or history of otologic disease [18,19,20]. However, the outcomes were not sufficiently homogeneous to allow a robust pooled estimate.

Therefore, ETD and middle-ear dysfunction were retained as secondary narrative outcomes rather than as a main quantitative endpoint.

Tomonaga et al. evaluated children with OME, nasal allergy, and community controls using allergy testing, impedance audiometry, and Eustachian tube function testing. Nasal allergy was more frequent among children with OME, while OME was more frequent among children with nasal allergy than among controls. Eustachian tube dysfunction following intranasal histamine challenge was also more frequent in allergy-associated groups. Because the study combined prevalence, provocation, and functional outcomes, it was retained only in the narrative synthesis. The more complete 1988 English-language report was used, while the overlapping 1987 Japanese-language publication was excluded [29].

### 3.7. Qualitative and Mechanistic Evidence

Several studies were retained for qualitative synthesis because they were clinically relevant but unsuitable for quantitative pooling. These included OME-only referral cohorts, adenoid hypertrophy/OME mixed cohorts, treatment-response studies, mechanistic studies, and studies assessing allergic inflammation or middle-ear dysfunction without a clean comparator group [21,22,23,24,25,26,27,28].

OME referral-clinic studies reported high prevalences of allergic rhinitis, asthma, eczema, or positive skin-prick testing among children with chronic or recurrent OME, but these studies lacked non-OME comparator groups and were therefore not included in the main meta-analysis [21]. Other studies examined allergic inflammation, middle-ear effusion mediators, or Eustachian tube physiology and were used to support mechanistic interpretation rather than quantitative risk estimation [24,25,26,27,28].

Adult or mixed-age studies evaluating atopy and abnormal tympanometry were also considered relevant for the broader concept of middle-ear dysfunction, but they were not pooled with pediatric OME studies because their outcomes were not equivalent to clinically diagnosed OME [23].

### 3.8. Risk of Bias

Risk of bias was assessed for the 10 comparative studies contributing to the quantitative syntheses or adjusted sensitivity interpretation using design-specific Joanna Briggs Institute critical appraisal tools. Two studies were judged to have low risk of bias, five moderate risk, one moderate-to-high risk, and two high risk. The most frequent methodological concerns were incomplete adjustment for confounding, selection of cases and controls from different source populations, referral-based recruitment, small sample size, and insufficiently standardized or selectively applied allergy assessment.

Studies with objective assessment of OME, clearly defined allergic rhinitis or IgE sensitization, comparable study groups, and appropriate multivariable adjustment were judged to have lower risk of bias. Higher risk judgments were assigned to studies with small or highly selected samples, limited control of confounding, differential or incomplete allergy testing, and less representative case or control selection. A summary of the overall judgments is presented in Table 3, and the detailed item-level JBI assessments are provided in Supplementary Table S3.

## 4. Discussion

### 4.1. Main Findings

In this systematic review and meta-analysis, allergic rhinitis/rhinitis was significantly associated with pediatric otitis media with effusion. The primary random-effects analysis showed that children with allergic rhinitis or rhinitis had higher odds of OME than children without allergic rhinitis/rhinitis. The pooled estimate was OR = 3.73, with a 95% confidence interval of 1.79–7.74. Although heterogeneity was moderate to substantial, the direction of effect was generally consistent across the included studies [9,10,11,12,13,14].

The expanded sensitivity analysis, which added the recent community-based study by Adekanye et al., showed a similar result. The pooled estimate remained significant, with OR = 3.46, 95% CI 1.92–6.23, and heterogeneity decreased slightly [15]. This supports the stability of the overall association. At the same time, the Adekanye study deserves a cautious interpretation because allergic rhinitis was clinically defined and did not remain an independent predictor of OME after multivariable adjustment [15].

A separate exploratory quantitative synthesis showed that atopy or IgE sensitization was also associated with OME, with OR = 5.45, 95% CI 1.33–22.35 [16,17]. This finding is clinically relevant, but it should not be overinterpreted. Only two studies were included in this analysis, and heterogeneity was high. Therefore, the result supports a possible association between allergic sensitization and OME, but it does not provide strong evidence of causality.

The evidence regarding allergic rhinitis and Eustachian tube dysfunction or broader middle-ear dysfunction was more heterogeneous. Studies used different outcomes, including ETDQ-7, tympanometry, tubomanometry, middle-ear pressure, abnormal tympanogram patterns, mastoid pneumatization, and otologic history [19,20,23,27,28]. For this reason, ETD and middle-ear dysfunction were not pooled quantitatively.

### 4.2. Clinical and Methodological Heterogeneity

The primary meta-analysis showed substantial heterogeneity (I^2^ = 69.7%), which likely reflects meaningful differences in study populations, exposure definitions, outcome ascertainment, and study setting. Allergic rhinitis was variably identified using symptom questionnaires, clinical examination, nasal cytology, skin-prick testing, specific IgE assays, or combinations of symptoms and objective sensitization testing [9,10,11,12,13,14,15,18]. Consequently, some studies evaluated strictly defined allergic rhinitis, whereas others may have included children with nonspecific or non-allergic rhinitis. OME definitions also differed, ranging from otoscopic findings combined with type B tympanometry to broader criteria that included type C or C2 tympanograms, chronicity requirements, ventilation-tube status, absent acoustic reflexes, or conductive hearing loss [9,10,11,12,13,14,15,16,17,18]. These variations may have contributed to exposure and outcome misclassification and limit direct comparability across studies.

Formal subgroup analysis or meta-regression was considered but was not performed because the primary synthesis included only six studies, and the number of studies within each diagnostic or methodological category was too small to provide stable and interpretable estimates. In particular, grouping studies according to objective versus symptom-based allergic rhinitis assessment, type B versus type B/C OME definitions, or community-based versus referral-clinic recruitment would have resulted in subgroups containing only one or two studies. Such analyses would have been underpowered and potentially misleading. The sources of heterogeneity were therefore examined qualitatively and are summarized in Table 4.

### 4.3. Confounding and Interpretation of Crude Estimates

Most study-level effects included in the quantitative syntheses were calculated from crude 2 × 2 data. Therefore, residual confounding by age, adenoid hypertrophy, recurrent upper respiratory tract infections, daycare attendance, tobacco-smoke exposure, previous acute otitis media, socioeconomic factors, and other determinants of pediatric OME cannot be excluded. The pooled odds ratios should consequently be interpreted as associations rather than causal effect estimates.

The influence of adjustment was not uniform across the available studies. In the community-based study by Adekanye et al., allergic rhinitis was associated with OME in the unadjusted analysis, but it was not retained as an independent predictor after adjustment for age and sex [15]. Conversely, Kreiner-Møller et al. reported that allergic rhinitis remained associated with OME after adjustment for animal and tobacco-smoke exposure, paternal atopy, household income, older siblings, sex, and previous acute otitis media episodes [18]. Chantzi et al. also identified IgE sensitization and selected respiratory allergy symptoms as independent predictors in multivariable analysis [16]. Together, these findings suggest that confounding may partly explain the magnitude of some crude associations, although the association may persist when allergic rhinitis or sensitization is defined objectively and relevant covariates are considered.

### 4.4. Biological Plausibility

Several mechanisms may explain the observed association between allergic rhinitis and OME. The Eustachian tube links the nasopharynx to the middle-ear cavity and is essential for ventilation, pressure regulation, and clearance. Allergic inflammation in the nose and nasopharynx may promote mucosal edema around the pharyngeal opening of the Eustachian tube, impair tubal opening, and contribute to negative middle-ear pressure [4,5].

The concept of a continuous upper-airway inflammatory pathway is also relevant. Yu et al. described several mechanisms through which allergic inflammation may contribute to ETD, including Th2-mediated inflammation, mucosal swelling, impaired mucociliary clearance, surfactant alterations, and extension of inflammation from the nasopharynx toward the Eustachian tube [4]. These mechanisms are compatible with the association observed in the present meta-analysis.

At the same time, the mechanism is unlikely to be simple or universal. Doyle et al. investigated whether the middle ear could behave as an allergic “shock organ” in a passively sensitized rhesus monkey model, but allergen challenge did not induce OME [7]. Fireman also emphasized that intranasal antigen or histamine challenge may induce transient Eustachian tube obstruction without necessarily producing middle-ear effusion [5]. These findings support a more cautious interpretation: allergic inflammation may contribute to OME in susceptible children, but it is unlikely to be sufficient as an isolated cause in all cases.

This interpretation fits the clinical reality of OME. Age, adenoid hypertrophy, viral infections, daycare exposure, smoke exposure, craniofacial factors, and previous acute otitis media may all influence OME risk. Allergic rhinitis may interact with these factors rather than act independently in every patient.

Current allergy nomenclature recognizes that allergic disease is not restricted to systemic IgE-mediated mechanisms. Hypersensitivity reactions may involve antibody-mediated, cell-mediated, tissue-driven, and mixed immune pathways, and allergic rhinitis may include endotypes extending beyond the classical IgE-dominant response [30]. Accordingly, studies relying exclusively on skin-prick testing or serum-specific IgE may not capture the full spectrum of immune-mediated rhinitis phenotypes. None of the studies included in the present quantitative syntheses systematically evaluated non-IgE-mediated allergic rhinitis using validated mechanistic or provocation-based methods. Its potential contribution to ETD or OME therefore remains uncertain and warrants further investigation.

### 4.5. Eustachian Tube Dysfunction and Middle-Ear Dysfunction

The narrative ETD findings support biological plausibility, but they should be interpreted separately from OME. Ma et al. evaluated patients with house dust mite allergic rhinitis using ETDQ-7, tympanometry, nasal endoscopy, and tubomanometry, and found evidence of altered Eustachian tube function in allergic rhinitis [19]. Adams et al., using NHANES data, reported associations between rhinitis and otologic history in adolescents, although not necessarily with abnormal tympanometry at the time of assessment [20].

These studies are relevant because they suggest that allergic rhinitis may affect middle-ear physiology even when OME is not the immediate endpoint. However, ETD is not measured uniformly across studies. Symptoms, tympanometric patterns, tubomanometry results, middle-ear pressure, and tympanostomy history are related but not identical outcomes. Pooling these data would risk producing a statistically neat but clinically weak result. For that reason, we treated ETD and middle-ear dysfunction as narrative outcomes.

### 4.6. Clinical Implications

The results support a phenotype-based approach rather than a universal allergy-based explanation for OME. Children with persistent allergic rhinitis, nasal obstruction, recurrent ear symptoms, suspected hearing loss, speech delay, or abnormal otoscopy may benefit from tympanometric evaluation and closer ENT follow-up.

However, this review does not show that all children with OME should receive allergy-directed treatment. OME remains multifactorial, and most included studies were observational. In addition, spontaneous resolution is common, which makes treatment-response studies difficult to interpret without adequate controls [1]. Allergy treatment is clinically appropriate when allergic rhinitis is present, but OME management should still follow otologic principles, including watchful waiting when appropriate, hearing evaluation, tympanometric monitoring, and surgical referral when indicated.

A practical implication is that pediatric ENT and allergy/immunology should not work in isolation in selected cases. Children with persistent OME and clear allergic rhinitis symptoms may require both middle-ear assessment and proper rhinitis phenotyping. This does not mean that allergy explains every case of OME, but it may identify a subgroup in whom nasal inflammation contributes to middle-ear dysfunction.

### 4.7. Strengths

This review has several strengths. It provides an updated synthesis of eligible evidence published through 2024, identified through literature searches updated to 8 June 2026. It also distinguishes clinically defined allergic rhinitis/rhinitis from atopy or IgE sensitization, thereby improving clinical and mechanistic interpretability.

Another strength is the conservative handling of heterogeneous outcomes. OME, ETD, abnormal tympanometry, and broader middle-ear dysfunction were not treated as interchangeable endpoints. ETD-related evidence was synthesized narratively because of substantial variability in study populations, diagnostic tools, and outcome definitions.

The review also used transparent study-level data extraction, separate quantitative syntheses for distinct exposure constructs, design-specific JBI critical appraisal tools, and an expanded sensitivity analysis. Inclusion of an additional community-based study resulted in only a limited change in the crude pooled estimate. However, because the adjusted association in that study was not statistically significant, the sensitivity analysis was interpreted as demonstrating numerical stability of the unadjusted estimate rather than confirmatory evidence of an independent association.

Finally, reports with unverifiable bibliographic information and studies without sufficiently reliable or extractable comparative data were not forced into the quantitative synthesis.

### 4.8. Limitations

This review has several limitations related both to the available evidence and to the review process. First, the included evidence was predominantly observational, with most studies using case–control or cross-sectional designs. Consequently, temporal relationships cannot be established, causality cannot be inferred, and the pooled associations should not be interpreted as causal effects.

Substantial clinical and methodological heterogeneity was present across the included studies. Definitions of allergic rhinitis, rhinitis, atopy, and IgE sensitization varied considerably. Exposure ascertainment ranged from symptom-based questionnaires and clinical assessment to nasal cytology, skin-prick testing, specific IgE measurement, or composite definitions requiring both symptoms and objective sensitization. Similarly, OME definitions differed across studies. Some studies required otoscopic findings combined with type B tympanograms, whereas others also included type C or C2 tympanograms, persistent effusion criteria, absent acoustic reflexes, conductive hearing loss, or ventilation-tube status. These differences may have introduced exposure and outcome misclassification and reduced direct comparability among studies.

Control of confounding was also inconsistent. Age, adenoid hypertrophy, recurrent upper respiratory tract infections, daycare attendance, tobacco-smoke exposure, previous acute otitis media, socioeconomic factors, and referral patterns may influence the occurrence of OME. Although some studies reported multivariable analyses, most estimates included in the quantitative syntheses were calculated from crude 2 × 2 data. Residual confounding therefore cannot be excluded and may partly account for the magnitude of the observed associations.

The number of studies available for each quantitative synthesis was limited. This precluded reliable subgroup analyses or meta-regression according to factors such as objective versus symptom-based allergic rhinitis assessment, type B versus type B/C tympanometric definitions, age group, geographic region, or community-based versus referral-clinic recruitment. Such analyses would have produced very small subgroups and unstable estimates. The exploratory atopy/IgE sensitization meta-analysis included only two studies and showed substantial heterogeneity; its pooled estimate should therefore be interpreted cautiously.

Formal assessment of small-study effects or publication bias was not performed because the number of studies in each meta-analysis was too small for funnel-plot asymmetry or statistical testing to be informative. Selective publication of positive associations nevertheless remains possible. In addition, no formal GRADE or other certainty-of-evidence framework was applied, and confidence in the findings was therefore evaluated qualitatively on the basis of study design, risk of bias, heterogeneity, precision, and consistency.

Risk-of-bias appraisal was performed for the 10 comparative studies contributing to the quantitative syntheses or adjusted sensitivity interpretation. Narrative-only mechanistic, descriptive, non-comparative, and treatment-response studies were used for contextual interpretation and were not included in the formal JBI appraisal. Conclusions based on these narrative studies should consequently be regarded as supportive and hypothesis-generating rather than as evidence of a quantified independent association.

The review process also had limitations. Initial screening, eligibility assessment, and data extraction were performed by the first author, with uncertain records and ambiguous information discussed with the senior authors. Although this approach provided adjudication of unclear cases, the absence of fully independent duplicate screening and data extraction may have increased the risk of study-selection or data-extraction error. Risk-of-bias assessment, by contrast, was conducted independently by two reviewers.

Finally, the review was not prospectively registered, and no public protocol was available. Although eligibility, extraction, and synthesis procedures were defined in a working review plan before the final analyses, the absence of prospective registration limits independent verification of whether methodological decisions were modified during the review process. These limitations indicate that the findings should be interpreted as evidence of an association between allergic disease and pediatric OME in selected populations, rather than as proof of a universal or causal relationship.

### 4.9. Future Research

Future studies should use standardized definitions of both allergic rhinitis and OME. Ideally, allergic rhinitis should be diagnosed using compatible nasal symptoms, evidence of sensitization, and relevant allergen exposure. OME should be confirmed using otoscopy and tympanometry, with clear separation between type B tympanograms, type C tympanograms, negative middle-ear pressure, and persistent effusion.

Future research should also adjust for major confounders, including age, adenoid hypertrophy, recurrent upper respiratory infections, daycare attendance, smoke exposure, previous acute otitis media, and craniofacial or developmental risk factors. For ETD, studies should combine validated symptom scores with objective measures such as tympanometry, tubomanometry, sonotubometry, or pressure-equilibration testing.

Clinical trials of allergy-directed treatment should focus on children with clearly defined allergic rhinitis phenotypes and should use objective middle-ear outcomes. Without this type of design, it will remain difficult to distinguish true treatment effect from spontaneous resolution or nonspecific improvement.

## 5. Conclusions

This systematic review and meta-analysis found a significant association between allergic rhinitis/rhinitis and pediatric otitis media with effusion. The crude pooled estimate changed only modestly after inclusion of an additional community-based study; however, the predominance of unadjusted observational data means that residual confounding cannot be excluded.

Atopy and IgE sensitization also appear to be associated with OME, although this evidence is based on only two studies and is characterized by substantial heterogeneity. These findings should therefore be regarded as exploratory rather than definitive.

The available evidence supports considering allergic disease as a potential contributing factor in selected pediatric OME phenotypes, particularly when persistent nasal symptoms coexist with recurrent ear complaints, suspected hearing loss, speech delay, abnormal otoscopy, or abnormal tympanometry. However, the findings do not support treating all pediatric OME as allergy-driven disease.

A phenotype-based approach involving both otolaryngology and allergy/immunology may improve clinical assessment in selected cases, but allergy-directed treatment should complement rather than replace standard OME management. Future prospective studies should use standardized definitions of allergic rhinitis, atopy, OME, and ETD, apply consistent objective diagnostic criteria, adequately adjust for major confounders, and evaluate both IgE-mediated and non-IgE-mediated rhinitis endotypes.

## Figures and Tables

**Table 1:** Characteristics of studies included in the systematic review and synthesis.

Study	Country	Design	Population	Exposure	Outcome	Assessment/Diagnostic Method	Role in Synthesis
Pau and Ng, 2016 [9]	Hong Kong	Cross-sectional	Children aged 4–12 years	Allergic rhinitis	OME	Pneumatic otoscopy and portable tympanometry	Primary AR/rhinitis–OME meta-analysis
Fasunla et al., 2017 [10]	Nigeria	Case–control	Children aged 2–7 years	Allergic rhinitis	OME	AR diagnosed by symptoms and nasal cytology; OME by Jerger tympanometric patterns	Primary AR/rhinitis–OME meta-analysis
Sharifian et al., 2019 [11]	Iran	Case–control	37 children with OME and 52 controls	Allergic rhinitis	OME	OME diagnosed by otoscopy and/or type B tympanogram; AR by clinical assessment and allergy testing where indicated	Primary AR/rhinitis–OME meta-analysis
Caffarelli et al., 1998 [12]	Italy	Case–control	172 children with OME and 200 controls	Allergic rhinitis/atopic symptoms	OME	OME by otoscopy and type B/C tympanograms; allergic disorders assessed by questionnaire, clinical examination, and skin-prick testing	Primary AR/rhinitis–OME meta-analysis
Yeo et al., 2007 [13]	South Korea	Prospective case–control	123 children with chronic OME and 141 controls	Allergic rhinitis	Chronic OME	OME by otoscopy and tympanometry; AR by history, examination, skin testing and/or MAST-CLA	Primary AR/rhinitis–OME meta-analysis
Kayhan et al., 2002 [14]	Turkey	Case–control	22 children with OME and 21 controls	Allergic rhinitis	OME	OME by physical examination and tympanometry; AR by symptoms, IgE, skin-prick testing and nasal smear	Primary AR/rhinitis–OME meta-analysis
Adekanye et al., 2024 [15]	Nigeria	Community cross-sectional	320 children aged 1–10 years	Clinically defined allergic rhinitis	OME	AR by rhinological symptoms plus allergy/family/asthma history; OME by otoscopy and tympanometry	Expanded sensitivity analysis
Kreiner-Møller et al., 2012 [18]	Denmark	Prospective birth cohort	COPSAC children assessed at 6 years	Allergic rhinitis	OME	AR defined by symptoms, sensitization and relevant exposure; OME by otoscopy and tympanometry	Adjusted-OR/narrative sensitivity
Chantzi et al., 2006 [16]	Greece	Case–control	88 children with OME and 80 controls, aged 1–7 years	IgE sensitization	OME	OME by symptoms, otoscopy and tympanometry; sensitization by SPT and/or CAP-FEIA	Atopy/IgE–OME meta-analysis
Martines et al., 2010 [17]	Italy	School cross-sectional screening	310 primary school children aged 5–6 years	Atopy by SPT	OME	OME by pneumatic otoscopy, type B/C tympanogram, absent acoustic reflex and conductive hearing loss	Atopy/IgE–OME meta-analysis
Ma et al., 2020 [19]	China	Prospective controlled cross-sectional study with treatment follow-up	59 adults with house dust mite allergic rhinitis and 59 controls	House dust mite allergic rhinitis	ETD/middle-ear dysfunction	ETDQ-7, tympanography, nasal endoscopy and tubomanometry	ETD narrative synthesis
Adams et al., 2023 [20]	USA	NHANES cross-sectional	Adolescents aged 12–19 years	Allergic and non-allergic rhinitis	ETD-related history and tympanometry	Serum IgE, rhinitis classification, tympanometry and otologic history	ETD narrative synthesis
Alles et al., 2001 [21]	United Kingdom	Referral-clinic observational study	209 children aged 3–8 years with chronic/recurrent OME	Atopic disorders/allergic rhinitis	Chronic/recurrent OME	Allergy history, examination, nasal smear and SPT	Qualitative synthesis only
Bemanian et al., 2020 [22]	Iran	Prospective cross-sectional/treatment-response study	122 children with adenoid hypertrophy and/or OME	Atopy/allergic symptoms	AHT and OME symptoms	OME by otoscopy, audiometry and tympanometry; allergy assessment by symptoms, IgE and SPT in selected patients	Qualitative synthesis only
Kaymakçı et al., 2015 [23]	Turkey	Prospective controlled study	33 atopic adults and 30 non-atopic controls	Atopy	Abnormal tympanometry/mastoid pneumatization	Atopy by SPT; tympanometry and 3D temporal CT mastoid volume measurement	Secondary qualitative ETD/middle-ear dysfunction synthesis
Norhafizah et al., 2020 [24]	Malaysia	Observational study	Children with OME	Allergic rhinitis	OME	Otologic and audiologic assessment as reported	Qualitative synthesis only
Hurst, 1996 [25]	USA	Mechanistic/clinical observational study	Patients with persistent OME	Atopy/allergy	OME and middle-ear inflammation	Skin testing, effusion markers and mucosal biopsy assessment	Mechanistic qualitative synthesis
Boedts et al., 1984 [26]	Belgium	Mechanistic observational study	Patients with OME/secretory otitis media	Atopic allergy/IgE	Middle-ear effusion	IgE assessment in effusion and serum	Mechanistic qualitative synthesis
Knight et al., 1992 [27]	USA	Seasonal exposure study	Patients with seasonal allergic rhinitis	Seasonal allergic rhinitis/pollen exposure	ETD/middle-ear pressure	Eustachian tube function and middle-ear pressure assessment	ETD qualitative synthesis
Osur et al., 1989 [28]	USA	Small seasonal exposure study	Children with ragweed hay fever	Ragweed allergic rhinitis	ETD	Eustachian tube obstruction/function assessment	ETD qualitative synthesis
Tomonaga et al., 1988 [29]	Japan	Clinical observational study	Children with OME, nasal allergy, and community controls	Nasal allergy diagnosed using clinical examination and multiple allergy tests	OME, tympanometric abnormalities, and Eustachian tube function	Otoscopy, audiometry, tympanometry, allergy testing, and Eustachian tube function tests	Narrative synthesis only; overlapping 1987 report excluded

AR, allergic rhinitis; AHT, adenoid hypertrophy; CAP-FEIA, fluorescence enzyme immunoassay; ETD, Eustachian tube dysfunction; ETDQ-7, Eustachian Tube Dysfunction Questionnaire-7; IgE, immunoglobulin E; MAST-CLA, multiple allergosorbent chemiluminescent assay; NHANES, National Health and Nutrition Examination Survey; OME, otitis media with effusion; SPT, skin-prick test.

**Table 2:** Quantitative synthesis data and study-level effect estimates.

Study	Analysis	Exposure	Outcome	a	b	c	d	OR	95% CI	Weight RE (%)	Use/Caveat
Pau & Ng 2016 [9]	Primary AR/rhinitis–OME	Allergic rhinitis	OME	12	147	3	182	4.95	1.37–17.88	14.7	Patient-level full text
Fasunla et al., 2017 [10]	Primary AR/rhinitis–OME	Allergic rhinitis	OME	39	47	8	78	8.09	3.48–18.79	19.7	Patient-level full text
Sharifian et al., 2019 [11]	Primary AR/rhinitis–OME	Allergic rhinitis	OME	9	3	28	49	5.25	1.31–21.01	13.6	Case–control full text
Caffarelli et al., 1998 [12]	Primary AR/rhinitis–OME	Allergic rhinitis/rhinitis symptoms	OME	28	11	144	189	3.34	1.61–6.94	21.1	SPT positivity not pooled as AR
Yeo et al., 2007 [13]	Primary AR/rhinitis–OME	Allergic rhinitis	Chronic OME	35	34	88	107	1.25	0.72–2.17	23.2	Counts explicitly reported; non-significant study-level association.
Kayhan et al., 2002 [14]	Primary AR/rhinitis–OME	Allergic rhinitis	OME	5	1	17	20	5.88	0.62–55.38	7.6	Small study
Pooled random-effects	Primary AR/rhinitis–OME	-	-	-	-	-	-	3.73	1.79–7.74	-	I^2^ = 69.7%; *p* < 0.001
Adekanye et al., 2024 [15]	Expanded sensitivity AR/rhinitis–OME	Clinically defined allergic rhinitis	OME	12	33	32	243	2.76	1.30–5.88	-	Added in expanded sensitivity; adjusted association not significant
Pooled random-effects incl. Adekanye	Expanded sensitivity AR/rhinitis–OME	-	-	-	-	-	-	3.46	1.92–6.23	-	I^2^ = 63.7%; *p* < 0.001
Chantzi et al., 2006 [16]	Exploratory atopy/IgE–OME	IgE sensitization	OME	28	12	60	68	2.64	1.24–5.66	49.7	SPT and/or CAP-FEIA; adjusted OR 2.52
Martines et al., 2010 [17]	Exploratory atopy/IgE–OME	Atopy by SPT	OME	24	32	16	238	11.16	5.36–23.20	50.3	School screening; objective OME criteria
Pooled random-effects	Exploratory atopy/IgE–OME	-	-	-	-	-	-	5.45	1.33–22.35	-	I^2^ = 86.0%; *p* = 0.018

a = exposed participants with outcome; b = exposed participants without outcome; c = unexposed participants with outcome; d = unexposed participants without outcome. Individual odds ratios were calculated as OR = (a × d)/(b × c). Dashes indicate not applicable for pooled random-effects estimates. AR, allergic rhinitis; CAP-FEIA, fluorescence enzyme immunoassay; CI, confidence interval; IgE, immunoglobulin E; OME, otitis media with effusion; OR, odds ratio; RE, random effects; SPT, skin-prick test.

**Table 3:** Summary of the JBI risk-of-bias assessment for studies contributing to the quantitative synthesis or adjusted sensitivity interpretation.

Study	JBI Appraisal Tool	Key Methodological Limitations	Overall Risk of Bias
Pau and Ng, 2016 [9]	JBI Analytical Cross-Sectional	Allergic rhinitis was assessed mainly using clinical symptoms and questionnaire-based criteria; no multivariable adjustment was performed.	Moderate
Fasunla et al., 2017 [10]	JBI Case–Control	Cases and controls were recruited from different source settings; no adjusted analysis for relevant confounders was reported.	Moderate
Sharifian et al., 2019 [11]	JBI Case–Control	Small, selected sample; allergy testing was not uniformly applied to all participants; no multivariable adjustment was performed.	High
Caffarelli et al., 1998 [12]	JBI Case–Control	Referral-clinic cases and school-based controls differed in source population and age distribution; no adjusted analysis was reported.	Moderate-to-high
Yeo et al., 2007 [13]	JBI Case–Control	Chronic OME cases were referral-based and selected for ventilation tube insertion; no multivariable adjustment was performed.	Moderate
Kayhan et al., 2002 [14]	JBI Case–Control	Very small sample; allergy testing was applied selectively; methodological reporting and control of confounding were limited.	High
Adekanye et al., 2024 [15]	JBI Analytical Cross-Sectional	Allergic rhinitis was defined using symptom- and history-based criteria without objective sensitization testing; the adjusted association was not statistically significant.	Moderate
Chantzi et al., 2006 [16]	JBI Case–Control	Controls were hospital-based, although groups were matched, exposure and outcome assessment were objective, and multivariable adjustment was performed.	Low
Martines et al., 2010 [17]	JBI Analytical Cross-Sectional	Atopy and OME were objectively assessed, but no adjustment for relevant confounders was reported.	Moderate
Kreiner-Møller et al., 2012 [18]	JBI Analytical Cross-Sectional analysis within a prospective birth cohort	The cohort included children born to mothers with asthma, which may limit generalizability; some participants had missing or inconclusive OME assessments.	Low

Risk-of-bias judgments were based on design-specific JBI critical appraisal criteria and on the methodological importance of the identified limitations rather than on an unweighted numerical score. “Low” indicates no major methodological concerns likely to materially affect the reported association; “Moderate” indicates limitations that may influence the estimate but do not invalidate the study; “Moderate-to-high” indicates multiple important concerns with a greater potential to affect the findings; and “High” indicates major limitations in participant selection, exposure or outcome assessment, control of confounding, or statistical reporting. Detailed item-level judgments are provided in Supplementary Table S3. AR, allergic rhinitis; JBI, Joanna Briggs Institute; OME, otitis media with effusion.

**Table 4:** Diagnostic definitions of allergic rhinitis/atopy and otitis media with effusion, and their potential contribution to heterogeneity.

Study	AR/Atopy Definition and Objective Assessment	OME Definition and Assessment	Estimate Used	Main Potential Source of Heterogeneity
Pau and Ng, 2016 [9]	AR identified from compatible nasal symptoms using the ARIR questionnaire; no systematic SPT or specific-IgE confirmation.	Pneumatic otoscopy and portable tympanometry.	Crude 2 × 2 estimate.	Symptom/questionnaire-based AR definition and no multivariable adjustment.
Fasunla et al., 2017 [10]	Watery rhinorrhea plus ≥ 1 additional nasal symptom for ≥3–4 weeks; nasal cytology with ≥5 eosinophils/high-power field; no systematic SPT or specific IgE.	Jerger tympanometry; type B and C patterns considered compatible with OME.	Crude 2 × 2 estimate.	Broader OME definition including type C and AR confirmation based on nasal eosinophilia rather than allergen-specific testing.
Sharifian et al., 2019 [11]	Clinical AR diagnosis; SPT in all OME cases but only in controls suspected of AR; total IgE, blood eosinophils, and nasal cytology also assessed.	Otoscopy and/or type B tympanogram; persistent/recurrent OME or ventilation-tube candidates.	Crude 2 × 2 estimate.	Differential allergy testing and selection of more severe OME cases.
Caffarelli et al., 1998 [12]	Rhinitis and atopic symptoms assessed by questionnaire and clinical examination; SPT performed in all participants.	Clinical glue ear plus type B or C tympanograms; controls had type A and no OME history.	Crude estimate for clinical rhinitis; SPT positivity not pooled as AR.	Referral cases versus school controls, age imbalance, and inclusion of type C tympanograms.
Yeo et al., 2007 [13]	Compatible nasal symptoms plus positive MAST-CLA and/or skin testing; nasal provocation performed in the AR group.	Chronic OME by otoscopy and type B or C tympanometry; cases underwent ventilation-tube insertion.	Crude 2 × 2 estimate.	Selected surgical chronic-OME cohort, inclusion of type C, and no confounder adjustment.
Kayhan et al., 2002 [14]	AR based on rhinitis symptoms, total IgE, SPT, and nasal smear; SPT/smear used only in children with raised IgE or recurrent symptoms.	Physical examination and tympanometry.	Crude 2 × 2 estimate.	Selective allergy work-up, very small sample, and limited reporting.
Adekanye et al., 2024 [15]	At least two nasal/ocular symptoms plus personal/family allergy history or asthma; no SPT or specific-IgE confirmation.	Otoscopy and tympanometry in a community sample.	Crude estimate in sensitivity analysis; adjusted OR interpreted narratively.	Broad symptom/history-based AR definition; association weakened after adjustment for age and sex.
Chantzi et al., 2006 [16]	Primary exposure was IgE sensitization; respiratory allergy symptoms used standardized definitions; SPT and/or CAP-FEIA performed.	Symptoms, otoscopy, and type B or C tympanometry; duration > 1 month or recurrent OME.	Crude 2 × 2 estimate pooled; adjusted OR interpreted narratively.	Objective sensitization but broader type B/C OME definition and hospital-based controls.
Martines et al., 2010 [17]	Atopy defined by positive SPT during school screening.	Persistent effusion ≥ 3 months plus pneumatic otoscopy, type B/C tympanogram, absent acoustic reflex, and conductive hearing loss > 25 dB.	Crude 2 × 2 estimate.	Strict composite OME definition but no adjustment for confounders.
Kreiner-Møller et al., 2012 [18]	AR required symptoms, specific-IgE sensitization, and relevant allergen exposure; non-allergic rhinitis and asymptomatic sensitization analyzed separately.	Otoscopy plus type B or C2 tympanogram (<−200 dPa); children with tubes classified as OME.	Adjusted OR used for narrative sensitivity interpretation.	Most specific AR definition and extensive adjustment, but high-risk birth cohort and broader OME classification including C2/tubes.

Definitions and diagnostic thresholds varied across studies. Exposure ascertainment ranged from symptom-based allergic rhinitis to objective SPT or specific-IgE sensitization and, in one study, a composite definition requiring symptoms, sensitization, and relevant allergen exposure. OME criteria also varied regarding inclusion of type C/C2 tympanograms, chronicity, ventilation-tube status, and additional audiological findings. These differences were considered important sources of clinical and methodological heterogeneity. AR, allergic rhinitis; CAP-FEIA, fluorescence enzyme immunoassay; IgE, immunoglobulin E; MAST-CLA, multiple allergosorbent chemiluminescence assay; OME, otitis media with effusion; SPT, skin-prick test.

## Data Availability

The extracted 2 × 2 data and study-level effect estimates used in the quantitative syntheses are reported in Table 2. Database search strategies, reports not retrieved, eligibility-stage exclusions, and detailed risk-of-bias assessments are provided in the Supplementary Materials. The working extraction workbook is available from the corresponding author on reasonable request.

## Supplementary Materials

The following supporting information can be downloaded at https://www.mdpi.com/article/10.3390/children13070892/s1, File S1: PRISMA 2020 Checklist; Table S1: Complete search strategies; Table S2: Reports not retrieved and reports excluded after eligibility assessment [31,32,33,34,35,36,37,38,39,40,41,42,43,44,45,46,47,48,49,50,51,52,53,54,55,56,57,58,59]; Table S3: Design-specific JBI critical appraisal of studies contributing to the quantitative syntheses or adjusted sensitivity interpretation.

## Abbreviations

The following abbreviations are used in this manuscript:

**Abbreviation**	**Definition**
AOM	Acute otitis media
AR	Allergic rhinitis
CI	Confidence interval
ENT	Ear, nose, and throat
ETD	Eustachian tube dysfunction
ETDQ-7	Eustachian Tube Dysfunction Questionnaire-7
IgE	Immunoglobulin E
MEP	Middle-ear pressure
NHANES	National Health and Nutrition Examination Survey
OME	Otitis media with effusion
OR	Odds ratio
PRISMA	Preferred Reporting Items for Systematic Reviews and Meta-Analyses
SPT	Skin-prick test
URTI	Upper respiratory tract infection
