# Supplementary material for: Allergic Rhinitis and Allergic Sensitization in Pediatric Otitis Media with Effusion: A Systematic Review and Meta-Analysis with Narrative Synthesis of Eustachian Tube Dysfunction
Source: Children (Basel). 2026 Jul 3;13(7):892. doi: 10.3390/children13070892 (PMC13406340; doi:10.3390/children13070892)
Supplement: Supplementary file 1 [file children-13-00892-s001.zip › File S1-PRISMA_2020_Checklist_FINAL_CONCORDANT.pdf]

PRISMA 2020 Checklist

Manuscript: Allergic Rhinitis and Allergic Sensitization in Pediatric Otitis Media with Effusion: A Systematic Review and Meta-Analysis with Narrative Synthesis of Eustachian Tube Dysfunction

Completed for submission to Children

Note: This checklist maps each PRISMA 2020 item to the corresponding manuscript section, table, figure, or supplementary material. Items not formally assessed are marked as not formally assessed/not applicable.

Section and topic	Item	PRISMA 2020 checklist item	Location where item is reported
TITLE	1	Identify the report as a systematic review.	Title page: article title identifies the manuscript as a "Systematic Review and Meta-Analysis".
ABSTRACT	2	See the PRISMA 2020 for Abstracts checklist.	Abstract: structured Background, Methods, Results, and Conclusions summary; reports eligibility, databases, synthesis approach, main pooled effects, heterogeneity, and interpretation.
INTRODUCTION – Rationale	3	Describe the rationale for the review in the context of existing knowledge.	Introduction: paragraphs describing OME burden, multifactorial pathogenesis, allergy/ETD biological plausibility, and heterogeneity of previous evidence.
INTRODUCTION – Objectives	4	Provide an explicit statement of the objective(s) or question(s) the review addresses.	End of Introduction: objective to update and refine evidence regarding allergic rhinitis, allergic sensitization, and pediatric OME, with ETD/middle-ear dysfunction as narrative outcomes.
METHODS – Eligibility criteria	5	Specify the inclusion and exclusion criteria for the review and how studies were grouped for the syntheses.	Methods 2.2 Eligibility Criteria explicitly distinguishes pediatric OME analyses from adult/mixed-age studies retained only for the secondary ETD/middle-ear dysfunction narrative synthesis; Methods 2.6 Outcomes; Results 3.2 Characteristics of Included Studies.
METHODS – Information sources	6	Specify all databases, registers, websites, organisations, reference lists and other sources searched or consulted to identify studies. Specify the date when each source was last searched or consulted.	Methods 2.3 Information Sources and Search Strategy: PubMed/MEDLINE, Scopus, Cochrane Library, manual reference screening; final search date 8 June 2026.
METHODS – Search strategy	7	Present the full search strategies for all databases, registers and websites, including any filters and limits used.	Methods 2.3 and Supplementary Table S1: database-specific search strategy; no date restriction applied.
METHODS – Selection process	8	Specify the methods used to decide whether a study met the inclusion criteria, including how many reviewers screened each record/report, whether they worked independently, and if applicable details of automation tools used.	Methods 2.4 Study Selection: title/abstract screening, retrieval, eligibility assessment, categorization, handling of overlapping datasets; screening by first author with uncertain records discussed with senior authors.
METHODS – Data collection process	9	Specify the methods used to collect data from reports, including how many reviewers collected data, whether they worked independently, processes for obtaining or confirming data, and details of automation tools used.	Methods 2.5 Data Extraction: data were extracted by A.M.P. using a predefined form; unclear or ambiguous information was discussed with the senior authors before final categorization. Extracted variables and the 2 × 2 data convention are reported.
METHODS – Data items	10a	List and define all outcomes for which data were sought. Specify whether all results compatible with each outcome domain in each study were sought, and if not the methods used to decide which results to collect.	Methods 2.6 Outcomes: primary AR/rhinitis–OME outcome, exploratory atopy/IgE–OME quantitative outcome, and ETD and middle-ear dysfunction narrative outcomes.

Section and topic	Item	PRISMA 2020 checklist item	Location where item is reported
METHODS – Data items	10b	List and define all other variables for which data were sought. Describe any assumptions made about missing or unclear information.	Methods 2.5 Data Extraction: first author, year, country, design, population, age, sample size, exposure/outcome definitions, diagnostic methods, comparators, 2 × 2 data, reported/adjusted ORs, methodological notes.
METHODS – Study risk of bias assessment	11	Specify the methods used to assess risk of bias in the included studies, including details of the tool(s) used, how many reviewers assessed each study and whether they worked independently, and if applicable details of automation tools used.	Methods 2.7 Risk of Bias Assessment: two reviewers (A.M.P. and R.C.) independently assessed the 10 comparative studies contributing to the quantitative syntheses or adjusted sensitivity interpretation using design-specific JBI Critical Appraisal Checklists. Narrative-only mechanistic, descriptive, non-comparative, and treatment-response studies were used contextually and were not included in the formal JBI appraisal. Disagreements were resolved through discussion. Item-level ratings and overall judgments are reported in Results 3.8, Table 3, and Supplementary Table S3.
METHODS – Effect measures	12	Specify for each outcome the effect measure(s) used in the synthesis or presentation of results.	Methods 2.8 Statistical Analysis: crude odds ratios with 95% confidence intervals; log odds ratios used for meta-analysis.
METHODS – Synthesis methods	13a	Describe the processes used to decide which studies were eligible for each synthesis.	Methods 2.2, 2.5, 2.6, and 2.8; Results 3.2: AR/rhinitis–OME primary meta-analysis, expanded sensitivity analysis, exploratory atopy/IgE–OME quantitative synthesis, and ETD/middle-ear dysfunction narrative synthesis.
METHODS – Synthesis methods	13b	Describe any methods required to prepare the data for presentation or synthesis, such as handling missing summary statistics or data conversions.	Methods 2.5 and 2.8: 2 × 2 extraction convention; OR = (a × d)/(b × c); no continuity correction required because no zero cells.
METHODS – Synthesis methods	13c	Describe any methods used to tabulate or visually display results of individual studies and syntheses.	Methods 2.8; Results 3.3–3.5; Tables 1–2; Figures 2–3 forest plots.
METHODS – Synthesis methods	13d	Describe any methods used to synthesize results and provide a rationale for the choice(s). If meta-analysis was performed, describe the model(s), method(s) to identify heterogeneity, and software package(s) used.	Methods 2.8: random-effects meta-analyses on log ORs using inverse-variance weighting; I^2 used for heterogeneity; rationale based on expected clinical and methodological heterogeneity. Calculations were performed using a predefined extraction workbook based on extracted 2 × 2 data, and forest plots were generated from study-level effect estimates.
METHODS – Synthesis methods	13e	Describe any methods used to explore possible causes of heterogeneity among study results.	Results 3.3–3.4, Table 4, and Discussion 4.2 and 4.8: qualitative exploration of heterogeneity based on exposure definitions, OME diagnostic criteria, study populations, recruitment settings, and confounder adjustment; expanded sensitivity analysis including Adekanye et al. (2024).
METHODS – Synthesis methods	13f	Describe any sensitivity analyses conducted to assess robustness of the synthesized results.	Methods 2.8; Results 3.4; Table 2; Figure 2: expanded sensitivity analysis including Adekanye et al. (2024).
METHODS – Reporting bias assessment	14	Describe any methods used to assess risk of bias due to missing results in a synthesis, arising from reporting biases.	Methods 2.8: formal small-study-effect or publication-bias testing was not performed because the number of studies in each quantitative synthesis was small.
METHODS – Certainty assessment	15	Describe any methods used to assess certainty or confidence in the body of evidence for an outcome.	No formal GRADE or certainty-of-evidence assessment was performed. Limitations and confidence in interpretation are discussed qualitatively in Discussion 4.8.

Section and topic	Item	PRISMA 2020 checklist item	Location where item is reported
RESULTS – Study selection	16a	Describe the results of the search and selection process, from the number of records identified in the search to the number of studies included in the review, ideally using a flow diagram.	Results 3.1 and Figure 1: 1201 records were identified, 245 duplicates were removed, 956 records were screened, 50 reports were sought, 4 were not retrieved, 46 were assessed for eligibility, 25 were excluded, and 21 studies were included in the qualitative or narrative synthesis; 9 contributed to at least one pooled quantitative synthesis and 1 additional study to adjusted narrative sensitivity interpretation.
RESULTS – Study selection	16b	Cite studies that might appear to meet the inclusion criteria but which were excluded, and explain why they were excluded.	Supplementary Table S2 individually lists the four reports not retrieved and the 25 reports excluded after eligibility assessment, together with one principal reason for each exclusion.
RESULTS – Study characteristics	17	Cite each included study and present its characteristics.	Results 3.2 Characteristics of Included Studies and Table 1: characteristics of the 21 studies included in the qualitative or narrative synthesis, including study design, population, exposure definition, outcomes, and synthesis role.
RESULTS – Risk of bias in studies	18	Present assessments of risk of bias for each included study.	Results 3.8, Table 3, and Supplementary Table S3 report design-specific JBI assessments for the 10 comparative studies contributing to the quantitative syntheses or adjusted sensitivity interpretation. The manuscript explicitly states that narrative-only mechanistic, descriptive, non-comparative, and treatment-response studies were used contextually and were not included in this formal appraisal.
RESULTS – Results of individual studies	19	For all outcomes, present for each study summary statistics for each group and an effect estimate and its precision, ideally using structured tables or plots.	Quantitative outcomes: Table 2 and Figures 2–3 provide study-level estimates, 95% CIs, and pooled results. Narrative outcomes are presented by study in Table 1 and Sections 3.6–3.7.
RESULTS – Results of syntheses	20a	For each synthesis, briefly summarise the characteristics and risk of bias among contributing studies.	Results 3.2 and 3.8; Tables 1 and 3; Discussion 4.1–4.3.
RESULTS – Results of syntheses	20b	Present results of all statistical syntheses conducted. If meta-analysis was done, present for each the summary estimate and its precision and heterogeneity.	Abstract; Results 3.3–3.5; Table 2; Figures 2–3: pooled ORs, 95% CIs, p values and I^2.
RESULTS – Results of syntheses	20c	Present results of all investigations of possible causes of heterogeneity among study results.	Results 3.3–3.4, Table 4, and Discussion 4.2: qualitative assessment of heterogeneity arising from differences in exposure definitions, OME diagnostic criteria, study populations, recruitment settings, and confounder adjustment; expanded sensitivity analysis including Adekanye et al. (2024).
RESULTS – Results of syntheses	20d	Present results of all sensitivity analyses conducted to assess robustness of the synthesized results.	Results 3.4; Table 2; Figure 2: expanded sensitivity analysis including Adekanye et al. (2024).
RESULTS – Reporting biases	21	Present assessments of risk of bias due to missing results for each synthesis assessed.	Not formally assessed. Methods 2.8 explains that small-study-effect or publication-bias testing was not performed because the number of studies in each quantitative synthesis was too small for such methods to be informative.
RESULTS – Certainty of evidence	22	Present assessments of certainty or confidence in the body of evidence for each outcome assessed.	Not formally assessed. No GRADE or other certainty-of-evidence framework was applied; the strength and limitations of the evidence are discussed qualitatively in Discussion 4.1 and 4.8 and in the Conclusions.

Section and topic	Item	PRISMA 2020 checklist item	Location where item is reported
DISCUSSION	23a	Provide a general interpretation of the results in the context of other evidence.	Discussion 4.1 Main Findings, 4.2 Clinical and Methodological Heterogeneity, 4.3 Confounding and Interpretation of Crude Estimates, and 4.4 Allergic Rhinitis, Sensitization, and Non-IgE-Mediated Mechanisms.
DISCUSSION	23b	Discuss any limitations of the evidence included in the review.	Discussion 4.8 Limitations.
DISCUSSION	23c	Discuss any limitations of the review processes used.	Methods 2.4–2.8 describe the review process, including initial screening, eligibility assessment, and data extraction by the first author, with uncertain records and ambiguous information discussed with the senior authors. Discussion 4.8 addresses the lack of prospective registration, the small number of studies, the inability to perform reliable subgroup or meta-regression analyses, the absence of formal publication-bias testing, the predominance of crude effect estimates, and the lack of fully independent duplicate screening and data extraction, which may have increased the risk of study-selection or data-extraction error.
DISCUSSION	23d	Discuss implications of the results for practice, policy, and future research.	Discussion 4.6 Clinical Implications; 4.9 Future Research; Conclusions.
OTHER INFORMATION – Registration and protocol	24a	Provide registration information for the review, including register name and registration number, or state that the review was not registered.	Methods 2.3 states that the review was not prospectively registered and that no formal protocol was made publicly available.
OTHER INFORMATION – Registration and protocol	24b	Indicate where the review protocol can be accessed, or state that a protocol was not prepared.	Methods 2.3 states that no formal review protocol was prospectively registered or made publicly available and that eligibility, extraction, and synthesis procedures were defined in the working review plan before the final analyses. No separate protocol is cited or publicly available.
OTHER INFORMATION – Registration and protocol	24c	Describe and explain any amendments to information provided at registration or in the protocol.	Not applicable because the review was not registered and no public protocol was available.
OTHER INFORMATION – Support	25	Describe sources of financial or non-financial support for the review, and the role of the funders or sponsors in the review.	Funding statement: “This research received no external funding.”
OTHER INFORMATION – Competing interests	26	Declare any competing interests of review authors.	Conflicts of Interest statement: authors declare no conflicts of interest.
OTHER INFORMATION – Availability of data, code and other materials	27	Report which of the following are publicly available and where they can be found: template data collection forms; data extracted from included studies; data used for analyses; analytic code; any other materials used in the review.	Table 2 reports the extracted 2 × 2 data and study-level effect estimates used in the quantitative syntheses. Supplementary Tables S1–S3 provide the search strategies, eligibility-stage exclusions, and item-level risk-of-bias assessments. The working extraction workbook is available from the corresponding author on reasonable request. No analytic code was used or made publicly available.

Abbreviations: AR, allergic rhinitis; CAP-FEIA, fluorescence enzyme immunoassay; CI, confidence interval; ETD, Eustachian tube dysfunction; IgE, immunoglobulin E; OME, otitis media with effusion; OR, odds ratio; PRISMA, Preferred Reporting Items for Systematic Reviews and Meta-Analyses; SPT, skin-prick test.
