# Supplementary material for: Allergic Rhinitis and Allergic Sensitization in Pediatric Otitis Media with Effusion: A Systematic Review and Meta-Analysis with Narrative Synthesis of Eustachian Tube Dysfunction
Source: Children (Basel). 2026 Jul 3;13(7):892. doi: 10.3390/children13070892 (PMC13406340; doi:10.3390/children13070892)
Supplement: Supplementary file 1 [file children-13-00892-s001.zip › Supplementary Table S3.pdf]

Supplementary Table S3. Design-specific JBI critical appraisal of studies contributing to the quantitative syntheses or adjusted sensitivity interpretation

Appraisal framework. Risk of bias was assessed independently by two reviewers (A.M.P. and R.C.), and disagreements were resolved through discussion. The 2020 JBI checklists for analytical cross-sectional and case-control studies were applied according to the design of the exposure-outcome comparison. Kreiner-Møller et al. [18] was appraised as an analytical cross-sectional analysis nested within a prospective birth cohort because allergic rhinitis and OME were assessed contemporaneously in the sixth year of life. Ratings were Yes (Y), No (N), Unclear (U), or Not applicable (NA). Overall judgments were based on the methodological importance of limitations, not on a numerical cut-off.

A. Analytical cross-sectional studies

Study	Q1	Q2	Q3	Q4	Q5	Q6	Q7	Q8	Overall RoB	Study-specific rationale
Pau & Ng, 2016 [9]	Y	Y	U	Y	N	N	Y	Y	Moderate	AR was assigned using a symptom questionnaire/clinical classification without systematic objective sensitization testing; OME was assessed by pneumatic otoscopy and tympanometry. No multivariable control of OME risk factors was reported.
Adekanye et al., 2024 [15]	Y	Y	N	Y	Y	Y	Y	Y	Moderate	Community sampling and objective OME assessment were strengths. AR was defined by symptoms plus allergy/family/asthma history without objective allergy testing. Multivariable regression was performed; the adjusted AR association was not significant.
Martines et al., 2010 [17]	Y	Y	Y	Y	N	N	Y	Y	Moderate	Atopy was measured by SPT and OME by a strict audiological/tympanometric algorithm. Important confounders were not formally identified or adjusted in the reported association.
Kreiner-Møller et al., 2012 [18]	Y	Y	Y	Y	Y	Y	Y	Y	Low	Nested cross-sectional analysis within a well-characterized birth cohort. AR required symptoms, sensitization, and relevant exposure; OME was objectively assessed. Logistic models adjusted for multiple prespecified confounders. Generalizability is limited by the high-risk cohort of children born to asthmatic mothers, but this is primarily an applicability issue.

Question key: Q1 Inclusion criteria clearly defined; Q2 Subjects and setting described; Q3 Exposure measured validly/reliably; Q4 Objective standard criteria for condition; Q5 Confounders identified; Q6 Strategies to address confounding; Q7 Outcome measured validly/reliably; Q8 Appropriate statistical analysis.

B. Case-control studies

Study	Q1	Q2	Q3	Q4	Q5	Q6	Q7	Q8	Q9	Q10	Overall RoB	Study-specific rationale
Fasunla et al., 2017 [10]	U	Y	Y	Y	Y	N	N	Y	Y	Y	Moderate	Cases and controls were similar in age and sex, but came from different recruitment settings (tertiary clinic versus staff school). AR and OME were assessed consistently; no multivariable adjustment was reported.
Sharifian et al., 2019 [11]	N	U	Y	U	N	N	N	Y	Y	Y	High	OME cases were highly selected children with persistent/recurrent disease requiring ventilation tubes. Control selection and matching were incompletely described. Allergy testing was performed selectively, creating differential verification/misclassification risk; no confounder adjustment was reported.
Caffarelli et al., 1998 [12]	N	N	Y	Y	Y	N	N	Y	Y	Y	Moderate-high	Cases were recruited from an OME referral center and controls from school health screening, with a meaningful age difference. Exposure and outcome assessment were reasonably standardized, but no matching or multivariable adjustment addressed selection and confounding.
Yeo et al., 2007 [13]	U	U	Y	Y	Y	N	N	Y	Y	Y	Moderate	Cases and controls were recruited in the same ENT setting and were similar in age, but explicit matching and source-population comparability were not fully described. AR and OME were objectively assessed; no adjusted association was reported.
Kayhan et al., 2002 [14]	Y	Y	Y	U	Y	N	N	Y	Y	U	High	Age- and sex-matched groups were used, but the sample was very small. Confirmatory allergy testing was restricted to children with elevated IgE or suggestive symptoms, and confounding was not addressed. Statistical reporting was limited.
Chantzi et al., 2006 [16]	Y	Y	Y	Y	Y	Y	Y	Y	Y	Y	Low	Cases and controls were matched and evaluated using the same objective methods. IgE sensitization was measured by SPT and/or CAP-FEIA. A priori power calculation and multivariable logistic regression addressed key confounders, although controls were hospital-based.

Question key: Q1 Groups comparable apart from disease status; Q2 Cases and controls appropriately matched; Q3 Same criteria used to identify cases and controls; Q4 Exposure measured validly/reliably; Q5 Exposure measured the same way in both groups; Q6 Confounders identified; Q7 Strategies to address confounding; Q8 Outcomes assessed validly/reliably in both groups; Q9 Exposure period meaningful; Q10 Appropriate statistical analysis.

C. Summary judgments for the quantitative syntheses or adjusted sensitivity interpretation

Study	Appraisal design	Overall risk of bias
Pau & Ng, 2016 [9]	Cross-sectional	**Moderate**
Fasunla et al., 2017 [10]	Case-control	**Moderate**
Sharifian et al., 2019 [11]	Case-control	**High**
Caffarelli et al., 1998 [12]	Case-control	**Moderate-high**
Yeo et al., 2007 [13]	Case-control	**Moderate**
Kayhan et al., 2002 [14]	Case-control	**High**
Adekanye et al., 2024 [15]	Cross-sectional	**Moderate**
Chantzi et al., 2006 [16]	Case-control	**Low**
Martines et al., 2010 [17]	Cross-sectional	**Moderate**
Kreiner-Møller et al., 2012 [18]	Cross-sectional analysis nested in birth cohort	**Low**

Interpretive note. The most common limitations were inadequate control of confounding, non-comparable recruitment sources for cases and controls, selective or symptom-based AR ascertainment, and small samples. The strongest evidence came from Chantzi et al. [12] and Kreiner-Møller et al. [18], which used objective exposure/outcome definitions and adjusted analyses.

Source of appraisal criteria: JBI Critical Appraisal Checklist for Analytical Cross Sectional Studies (2020) and JBI Critical Appraisal Checklist for Case Control Studies (2020).
