# Supplementary material for: Allergic Rhinitis and Allergic Sensitization in Pediatric Otitis Media with Effusion: A Systematic Review and Meta-Analysis with Narrative Synthesis of Eustachian Tube Dysfunction
Source: Children (Basel). 2026 Jul 3;13(7):892. doi: 10.3390/children13070892 (PMC13406340; doi:10.3390/children13070892)
Supplement: Supplementary file 1 [file children-13-00892-s001.zip › Supplementary_Table_S1_S2.pdf]

Supplementary Materials

Allergic Rhinitis and Allergic Sensitization in Pediatric Otitis Media with Effusion: A Systematic Review and Meta-Analysis with Narrative Synthesis of Eustachian Tube Dysfunction

Alina-Mihaela Petre, Romeo Costin, Ion Anghel

Table S1. Complete search strategies.

Database/source	Complete search strategy	Search date	Limits/filters
PubMed/MEDLINE	("allergic rhinitis" OR rhinitis OR allergy OR atopy OR "IgE sensitization") AND ("otitis media with effusion" OR OME OR "middle ear effusion" OR "Eustachian tube dysfunction" OR ETD OR tympanometry OR "middle-ear dysfunction")	8 June 2026	No date restriction
Scopus	TITLE-ABS-KEY ("allergic rhinitis" OR rhinitis OR allergy OR atopy OR "IgE sensitization") AND TITLE-ABS-KEY ("otitis media with effusion" OR OME OR "middle ear effusion" OR "Eustachian tube dysfunction" OR ETD OR tympanometry OR "middle-ear dysfunction")	8 June 2026	No date restriction
Cochrane Library	("allergic rhinitis" OR rhinitis OR allergy OR atopy OR "IgE sensitization") AND ("otitis media with effusion" OR OME OR "middle ear effusion" OR "Eustachian tube dysfunction" OR ETD OR tympanometry OR "middle-ear dysfunction")	8 June 2026	No date restriction
Manual reference screening	Reference lists of eligible articles and relevant reviews were screened for additional potentially eligible reports.	Completed 8 June 2026	Not applicable

Note: The database-specific search strings are reported as used. No date restriction was applied. Duplicate records were removed after export and before title/abstract screening.

Table S2. Reports not retrieved and reports excluded after eligibility assessment [31–59].

Stage	Report	Principal reason
Not retrieved	Ruggeri et al., 1990 - Relations between allergic rhinitis and otitis media with effusion [31]	Full report could not be retrieved and verified.
Not retrieved	Baeza-Bacab et al., 1987 - Tympanometric abnormalities in children with allergic rhinitis [32]	Full report could not be retrieved and verified.
Not retrieved	Bernstein, 1980- Otitis media with effusion: an allergic disease? [33]	Full report could not be retrieved and verified.
Not retrieved	Bluestone, 1978 - Eustachian tube function and allergy in otitis media [34]	Full report could not be retrieved and verified.
Excluded	Marimuthu et al., 2026 - Comparison of Middle Ear Function among Patients with Allergic Rhinitis and Healthy Participants [35]	Abstract-only record; a sufficiently detailed full report was not available for complete eligibility and methodological assessment.
Excluded	Mukherjee et al., 2026 - Silent Middle Ear Dysfunction in Patients with Persistent Allergic Rhinitis: A Case-Control Study [36]	Abstract-only record; a sufficiently detailed full report was not available for complete eligibility and methodological assessment.
Excluded	Bulut and Erdem, 2025 - Evaluation of Eustachian Tube Functions in Adult Allergic Rhinitis Patients [37]	Adult AR-only cohort without an AR-negative comparator and without a unique contribution beyond the predefined narrative synthesis.
Excluded	Tukur et al., 2022 - Allergic Rhinitis: An Indicator of Otitis Media with Effusion in Children [38]	Source and outcome data were insufficiently reliable for patient-level extraction or full methodological verification.
Excluded	Riaz et al., 2018 - Frequency of Otitis Media with Effusion in Children with Allergic Rhinitis [39]	AR-only prevalence cohort without a non-AR comparator and without a unique non-duplicative contribution.
Excluded	Klancnik et al., 2016 - The Association of Allergy and Otitis Media with Effusion in Children [40]	Insufficiently verifiable methodological detail and non-extractable comparative data.
Excluded	Sharma et al., 2016 - Middle Ear Dysfunction: Connection to Allergic Rhinitis [41]	Treatment-response study without an eligible independent exposure-outcome comparison.
Excluded	Ni et al., 2012 - Correlation between Otitis Media with Effusion and Allergic Rhinitis [42]	Insufficiently detailed and non-extractable report for eligibility and methodological assessment.
Excluded	Souter et al., 2009 - The Prevalence of Atopic Symptoms in Children with Otitis Media with Effusion [43]	OME-only prevalence study without an eligible external comparator and without unique non-duplicative evidence.
Excluded	Pelikan, 2009 - Audiometric Changes in Chronic Secretory Otitis Media Due to Nasal Allergy [44]	Nasal provocation/mechanistic design without an eligible conventional exposure-outcome comparison.
Excluded	Marseglia et al., 2008 - Increased Risk of Otitis Media with Effusion in Allergic Children Presenting with Upper-Airway Infection [45]	Abstract-only record; a sufficiently detailed full report was not available for complete eligibility and methodological assessment.
Excluded	Pelikan, 2007 - The Role of Nasal Allergy in Chronic Secretory Otitis Media [46]	Nasal provocation/mechanistic design without an eligible conventional exposure-outcome comparison.

Stage	Report	Principal reason
Excluded	Lazo-Saenz et al., 2005 - Eustachian Tube Dysfunction in Allergic Rhinitis [47]	Outcome reporting and study design were not sufficiently compatible with the predefined synthesis and did not provide extractable comparative data.
Excluded	Sente et al., 2001 - Allergic Rhinitis as a Possible Etiologic Cause of Eustachian Tube Dysfunction [48]	Insufficiently verifiable methodological detail and non-extractable comparative data.
Excluded	Suzuki, 2001 - The Relationship between Otitis Media with Effusion and Allergic Rhinitis [49]	Review/discussion article without an original eligible dataset.
Excluded	Suzuki and Mogi, 1998 - A Link between Allergic Rhinitis and Otitis Media with Effusion [50]	Review/background article without an original eligible dataset.
Excluded	Becker et al., 1991 - Recurrent Middle Ear Effusions and Allergy in Children [51]	Insufficiently verifiable methodological detail and non-extractable comparative data.
Excluded	Fidalgo Alvarez et al., 1990 - Eustachian Tube Dysfunction in Children with Allergic Rhinitis [52]	Insufficiently detailed report for complete methodological and outcome assessment.
Excluded	Bachert et al., 1989 - Secretory Otitis Media and Allergy in Children [53]	Special adenoid/SOM cohort without an eligible external comparator and without unique non-duplicative evidence.
Excluded	Tomonaga et al., 1987 - The Role of Type I Allergy in Otitis Media with Effusion [54]	Duplicate/overlapping report; the more complete 1988 English-language publication was retained for narrative synthesis.
Excluded	Umehara et al., 1985 - Role of Atopic Allergy in Otitis Media with Effusion: A Clinical Observation [55]	Historical broad atopy definition and insufficiently compatible/non-extractable comparative data.
Excluded	McMahan et al., 1981 - Chronic Otitis Media with Effusion and Allergy: Modified RAST Analysis [56]	Treatment/RAST cohort without a clean eligible exposure-outcome comparison.
Excluded	Ruokonen et al., 1981 - Secretory Otitis Media and Allergy [57]	Historical allergy construct and cytotoxic leukocyte testing not aligned with the predefined AR/IgE exposure definitions.
Excluded	Rahko et al., 1979 - The Occurrence of Secretory Otitis Media in Allergic Children [58]	Allergic cohort only, without a non-allergic comparator and without unique non-duplicative evidence.
Excluded	Fernandes et al., 1978 - Tympanometry in Children with Allergic Respiratory Disease [59]	Mixed allergy-clinic cohort without a non-allergic comparator; only limited abstract-level information was available.

Note: The 906 records excluded during title/abstract screening are reported in the PRISMA flow diagram and were not listed individually. One principal reason is reported for each eligibility-stage exclusion. The 1988 English-language Tomonaga report was retained in the narrative synthesis, whereas the overlapping 1987 report was excluded.

Supplementary References

1. Ruggeri, C.; Barberio, G.; Pajno ,G.B.; Putortì, A.; Morabito, L.; Pollicino, A.; Febbraro, R. Relations between allergic rhinitis and otitis media with effusion. The role of the Eustachian tube. *Minerva Pediatr*. **1990**, *42*, 481–483.

2. Baeza-Bacab, M.A.; Sienra-Monge, J.J.; Paredes-Novelo, M.C.; Shturman-Ellstein, R. Tympanometric abnormalities in children with allergic rhinitis. *Rev Alerg Mex*. **1987**, *34*, 35–38.
3. Bernstein, J.M. Otitis media with effusion: an allergic disease? *Compr Ther*. **1980**, *6*, 15–21.
4. Bluestone, C.D. Eustachian tube function and allergy in otitis media. *Pediatrics*. **1978**, *61*, 753–760.
5. Marimuthu, M.; Sharma, P.V.; Singh, R.; Aithal, V.U. Comparison of Middle Ear Function among Patients with Allergic Rhinitis and Healthy Participants. *Indian J Otolaryngol Head Neck Surg*. **2026**, *78*, 265–269.
6. Mukherjee, Y.; Saha, S.; Sarkar, A.; Kar, T.; Ashraf, A.; Sau, T. Silent Middle Ear Dysfunction in Patients with Persistent Allergic Rhinitis: A Case Control Study. *Indian J Otolaryngol Head Neck Surg*. **2026**, *78*, 1681–1687.
7. Bulut, O.; Erdem, D. Evaluation of Eustachian tube functions in adult allergic rhinitis patients using Eustachian tube dysfunction questionnaire-7. *B-ENT*. **2025**, *21*,19–23.
8. Tukur, A.R.; Mahmud, A.; Jibril, Y.N.; Jalo, R.I.; Salisu, A.D. Allergic rhinitis: An indicator of otitis media with effusion in children seen at aminu kano teaching hospital, Kano. *Niger J Clin Pract*. **2022**, *25*, 1725–1730.
9. Riaz, M.; Rashid, T.; Javaid, W.; Khan, M.F.; Fatima, M.; Jaffery, S. Frequency of Otitis Media with Effusion in Children with Allergic Rhinitis. *PJMHS*. **2018**. *12*.
10. Klancnik, M.; Grgec, M.; Lozić, B.; Sunara, D. The Association of Allergy and Otitis Media with Effusion in Children. *Paediatr Croat*. **2016**, *60*, 58–63.
11. Sharma, S.; Srivastava, A.; Sharma, C.M. Middle Ear Dysfunction: connection to allergic rhinitis. *Clinical Rhinology: An International Journal*. **2016**, *9*, 65–67.
12. Ni, K.; Li, X. Correlation analysis between the otitis media with effusion and allergic rhinitis in children with adenoidal hypertrophy. *J Clin Otorhinolaryngol Head Neck Surg*. **2012**, *26*, 884–886.
13. Souter, M.A.; Mills, N.A.; Mahadevan, M.; Douglas, G.; Ellwood, P.E.; Asher, M.I.; Clayton, T.O.; Douglas, R.G. The prevalence of atopic symptoms in children with otitis media with effusion. *Otolaryngol Head Neck Surg*. **2009**, *141*, 104–107.
14. Pelikan, Z. Audiometric Changes in Chronic Secretory Otitis Media Due to Nasal Allergy. *Otology & Neurotology*. **2009**, *30*, 868–875.
15. Marseglia, G.L.; Pagella, F.; Caimmi, D.; Caimmi, S.; Castellazzi, A.M.; Poddighe, D.; Klersy, C.; Ciprandi, G. Increased risk of otitis media with effusion in allergic children presenting with adenoiditis. *Otolaryngol Head Neck Surg*. **2008**, *138*, 572–575.
16. Pelikan, Z. The role of nasal allergy in chronic secretory otitis media. *Ann Allergy Asthma Immunol*. **2007**, *99*, 401–407.
17. Lazo-Sáenz, J.G.; Galván-Aguilera, A.A.; Martínez-Ordaz, V.A.; Velasco-Rodríguez, V.M.; Nieves-Rentería, A.; Rincón-Castañeda, C. Eustachian tube dysfunction in allergic rhinitis. *Otolaryngol Head Neck Surg*. **2005,** *132*, 626–629.
18. Sente, M.; Sente, R.; Puleva, K.; Milekić, N.K. Allergic rhinitis as a possible etiologic cause eustachian tube dysfunction. *Med Pregl*. **2001**, 54, 166–171.
19. Suzuki, M. The Relationship between Otitis Media with Effusion and Allergic Rhinitis. *Otorhinolaryngology*. **2001**, *94*, 299–303.
20. Suzuki, M.; Mogi, G. A link between allergic rhinitis otitis media with effusion. *Allergology International*. **1998**, *47*, 177–182.
21. Becker, S.; Koch, T.; Philipp, A. Allergic origin of recurrent middle ear effusion and adenoids in young children. *HNO*. **1991**, *39*, 182–184.
22. Fidalgo Alvarez, I.; Risueno, M.T.; Carrasco, J.A.G.; Fuente, J.M.A. Eustachian Tube Dysfunction in Children with Allergic Rhinitis. *Otolaryngology*.**1990**.
23. Bachert, C.; Keilmann, A.; Ganzer, U. Secretory Otitis Media and Allergy in Children. *Laryngorhinootologie*. **1989**, *68*, 201–203.
24. Tomonaga, K.; Kurono, Y.; Mogi, G. The role of type I allergy in otitis media with effusion. *Nihon Jibiinkoka Gakkai Kaiho*. **1987**, *90*, 1840–1848.
25. Umehara, T.; Mogi, G.; Maeda, S.; Fujiyoshi, T.; Kurono, Y.; Kawauchi, H.; Yoshimura, H. Role of atopic allergy in otitis media with effusion - A clinical observation. *Practica oto-rhino-laryngologica*. **1985**, *78*, 1051–1057.
26. McMahan, J.T.; Calenoff, E.; Croft, D.J.; Barenholtz, L.; Weber, L.D. Chronic otitis media with effusion and allergy: modified RAST analysis of 119 cases. *Otolaryngol Head Neck Surg*. **1981**, *89*, 427–431.
27. Ruokonen, J.; Holopainen, E.; Palva, T.; Backman, A. Secretory Otitis Media and Allergy. *Allergy*. **1981**, *36*, 59–68.
28. Rahko, T.; Koivikko, A.; Silvonniemi, P. The occurrence of secretory otitis media in allergic children. *Clin Otolaryngol Allied Sci*. **1979**, *4*, 267–270.
29. Fernandes, D.; Gupta, S.; Sly, R.M.; Frazer, M. Tympanometry in children with allergic respiratory disease. *Annals of Allergy*. **1978**, 40, 181–184.
